# What is the role of the practice nurse in the care of people living with dementia, or cognitive impairment, and their support person(s)?: a systematic review

**DOI:** 10.1186/s12875-020-01177-y

**Published:** 2020-07-13

**Authors:** Caroline Gibson, Dianne Goeman, Dimity Pond

**Affiliations:** 1 Faculty of Health and Medicine, School of Medicine and Public Health, University of Newcastle, Callaghan, Australia; 2 Central Clinical School, Monash University; Kolling Institute, the University of Sydney, Sydney, Australia

**Keywords:** Practice nurse, Primary health care nurse, Dementia, Cognitive impairment

## Abstract

**Background:**

The potential value of expanding the Practice Nurse role to include the recognition and management of dementia has been acknowledged. Practice Nurses are well-positioned to provide comprehensive dementia information and support so that people living with dementia are better equipped to self-manage their health and live well with dementia. The purpose of this review was to systematically examine published literature to identify existing and potential roles of Practice Nurse’s in the delivery of care to people affected by dementia and to describe the characteristics and effectiveness of nurse interventions in dementia models of care.

**Methods:**

The PRISMA statement guided the systematic review of the quantitative and qualitative evidence for roles and characteristics of the Practice Nurse in the delivery of dementia care. A comprehensive literature search of seven electronic databases and Google scholar identified relevant original research published in English between January 2000 and January 2019. Thirteen articles met the inclusion criteria and were extracted into the Covidence software for analysis.

**Results:**

The heterogeneity of the included studies purpose, design and outcomes measures and the diversity in health systems and primary care nurses scope of practice made it difficult to synthesise the findings and draw conclusions. The heterogeneity did, however, provide important insights into the characteristics of roles undertaken by nurses working in the general practice setting, which were potentially beneficial to people living with dementia and their support person. These included patient accessibility to the Practice Nurse, early recognition and management of cognitive changes, care management and collaboration with the General Practitioner. Limitations of the provision of dementia care by Practice Nurses included a lack of definition of the role, inadequate dementia specific training, time constraints and poor communication with General Practitioners.

**Conclusions:**

Embedding an evidence-based model that describes the role of the Practice Nurse in dementia care provision has the potential to increase early recognition of cognitive impairment and more appropriate primary care management of dementia.

**Systematic review registration:**

PROSPERO 2018 CRD42018088191.

## Introduction

Australian and international literature [1, 2] reveals a significant gap in the delivery of dementia care in the general practice setting. In one study, 66% of participants (people with memory concerns) reported that they would like a memory test and 81% reported that they would speak with their General Practitioner (GP) if they thought they had dementia [3]. However, despite people’s intent to report their concerns with their GP, there is a significant gap in the delivery of dementia care in the general practice setting [1]. Barriers to the identification, diagnosis and management of dementia are multiple and complex, and in some cases include a perception by the GP that nothing can be done and that support options are lacking [4]. Dementia is the second leading cause of death in Australia and currently more than 400 000 Australians are living with dementia (5). This number is expected to increase three-fold by 2056 [5]. Around 83% of all males with dementia and 71% of females with dementia live in the community [5] with 50 percent of dementia cases remaining undiagnosed [6]. When combining these figures with the approximately 200 000 unpaid care-givers involved in supporting a person living with dementia [5] a significant number of people are likely to be attending general practices and not having their health and social care needs met. Exploring new ways to improve the identification and management of dementia in the primary care setting is needed.

Approximately two thirds of Australian general practices employ a nurse [7] and nurse-led clinics are known to maximise patient health outcomes in primary care [8, 9]. The Practice Nurse (PN) is a primary health care nurse employed in General Practice. As described by the Australian Primary Health Care Nurse Association (APNA) the role of the PN can include women’s health, men’s health, aged care, chronic disease management, immunisation, wound management, health promotion and population health. Given that co-morbidity in people living with dementia is high [8, 10] the PN is likely to have established a therapeutic relationship with people with cognitive decline through routine primary care treatment, health assessment, chronic disease management and health promotion activities.

The potential value of expanding the PN role to include the recognition and management of dementia has been acknowledged [4, 11, 12]. However, there is limited research on the role of the PN in dementia care delivery in Australian or in international literature. A significant barrier to GP’s discussing dementia with their patients is the perception that nothing can be done and that support options are lacking [4]. Developing a model of dementia care that incorporates a flexible clinical pathway to guide the PN, along with a compendium of resources that can be used to draw upon additional knowledge to assist in providing appropriate care for people with dementia, could help to overcome these barriers. The PN could offer the GP a means of providing immediate support to patients and their families, following a discussion about dementia that includes a conversation about their concerns and referral on to further supports as needed.

In summary, a PN model of dementia care has the potential to assist with the identification of cognition concerns and understanding of the impact of dementia on the health and well-being of an individual. Such a model is not only likely to lead to increased identification of dementia but also to more appropriate primary care treatment, chronic disease management, and, care planning for people with existing or emerging cognitive impairment or dementia and the people supporting them.

There has been no systematic review of the evidence on the role of the PN in dementia care delivery to date, therefore the aim of this review is to examine published literature to investigate the Practice Nurse role in the delivery of care to people affected by dementia.

This paper systematically reviews published literature to answer the review questions:

1. What are the existing and potential roles performed by the PN in the care of people living with dementia or cognitive impairment and their informal caregivers in General Practice?
2. What are the characteristics of any existing nurse interventions that provide care to people living with dementia, or cognitive impairment, and their informal caregivers in the General Practice setting?

The 27 item PRISMA-P Checklist [13] was used to guide this systematic review. The checklist includes items deemed essential for systematic review reporting [14].

## Methods

### Eligibility criteria

All published literature that described a role in care of a person with dementia and/ or their caregiver performed by a nurse in a General Practice setting published between the dates 1 January 2000 and 1 January 2019 were eligible for inclusion. Studies were limited to those published in English language.

### Information sources

A search strategy was developed to identify published peer-reviewed studies describing the role of the PN in the care of people living with dementia, or cognitive impairment, and their informal caregivers in general practice.

Seven electronic databases (Cochrane Library, EMBASE, CINHAHL (EBSCO), OVID MEDLINE (PubMed), Scopus, INFORMIT HEALTH and PsycINFO) and Google Scholar were searched.

A review of the included paper’s reference lists and citations was undertaken to identify any additional studies that may not have been identified in the primary search.

### Search strategy

Original searches were carried out on the 24^th^ February, 2018. Automatic search strategies for all included electronic databases were set up with weekly email alerts to identify eligible studies published from the date of the original search to 1^st^ January 2019. Search terms used included:

1. Practice Nurse, Primary Health Care Nurse , Primary Care Nurse, General Practice Nurse, General Practice Nurse (MeSH Nurse)
2. Dementia, Cognitive impairment, Cognitive deficit, Alzheimer’s disease, Memory loss, Vascular dementia, Lewy body dementia, Frontotemporal dementia, Younger onset dementia (MeSH Dementia) Cognitive impairment, Cognitive deficit, Cognitive decline, Cognitive dysfunction (MeSH Cognitive dysfunction)

Example of a search query

Medline

1. (Practice Nurs* or Primary Health Care Nurs* or Primary Care Nurs* or General Practice Nurs* or GP Nurs*).af.
2. (Dementia, or Cognitive impairment or Cognitive dysfunction or cognitive deficit or cognitive decline or alzheimer* or memory impairment or memory loss).af.

### Study selection

All records from searches were retrieved in Endnote reference management software, and transferred to Covidence, the on-line standard production platform for Cochrane Reviews (https://www.covidence.org/home). Using Covidence, all records were independently screened for eligibility using the identified inclusion criteria by two authors (CG and DG). Any discrepancies were resolved by a consensus meeting with the third author (DP).

**Fig. 1:**
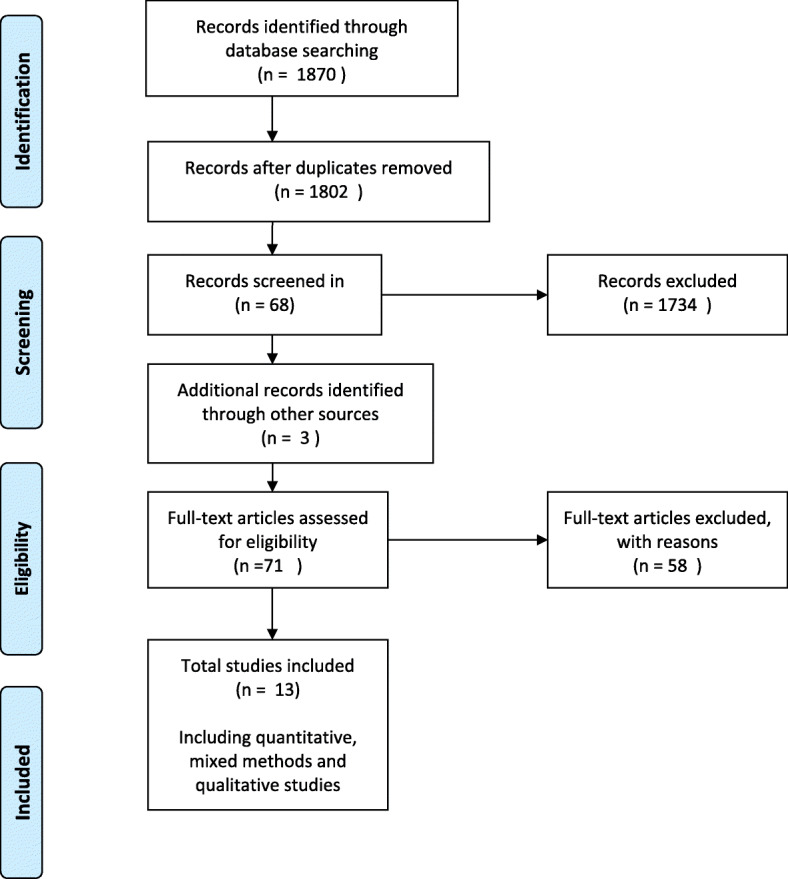
Study selection

The steps taken for paper selection were an initial screening for relevance using the titles of identified references. Papers considered to be irrelevant were removed from the selection process. A conservative approach was taken. Abstracts of remaining titles were reviewed based on inclusion criteria. The abstracts were coded relevant, irrelevant or unsure. The irrelevant papers were discarded from the selection process. Published papers were retrieved for abstracts categorised as relevant or unsure. The retrieved papers were then reviewed and those deemed as meeting the selection criteria were included in the systematic review (see Fig. 1).

Where the findings of a study have been published as separate papers due to the reporting of different outcome measures the paper with the most detailed analysis relevant to the aims of this systematic review was included. The other papers adding information to the paper included in this systematic review were described as supplementary papers.

### Data Collection processes

Data extraction for all study types included: author, year, country; aim; research design; instruments; sample and size; intervention type; analysis methods and outcomes. This information is described in Tables 5, 6, 7 and 8.

### Quality and risk of bias assessment

Two reviewers (CG, DG) independently assessed the studies for quality and risk of bias according to their specific study types. Any disagreements between the reviewers were resolved by discussion, with involvement of a third reviewer (DP).

Randomised Controlled Trial (RCT) studies were assessed for risk of bias using the Cochrane Risk of Bias Tool [44]. The CEBM Critical Appraisal tool [45] was used to assess the risk of bias in methodology, analysis and outcomes in cross-sectional studies. Mixed methods data was appraised using the Mixed Methods Appraisal Tool (MMAT) Version 2018 [46]. Risk of bias in qualitative studies was appraised using a tool based on the Critical Appraisal Skills Programme (CASP) Qualitative checklist [47]. The assessment criteria for each of the quality appraisal tools used is described in Tables 1, 2, 3 and 4.

**Table 1:** Risk of bias summary. Randomised controlled trials. Cochrane Risk of Bias Tool [44]

	Random sequence generation (selection bias)	Allocation concealment (selection bias)	Blinding participants and personnel for all outcomes (performance bias)	Blinding of outcome assessors for all outcomes	Incomplete outcome data for all outcomes (attrition bias)	Selective outcome reporting (reporting bias)	Other sources of bias
Callahan et al., 2006 [18]	+	+	?	+	+	+	+
Thyrian et al., 2017 [19]	+	-	-	-	-	+	+
Van den Dungen et al., 2016 [15]	+	-	-	?	-	+	+

- High risk of bias, + Low risk of bias, ? Unclear risk of bias

**Table 2:** Risk of bias summary. Qualitative studies. Based on the CASP Qualitative checklist [47]

Criteria										
Study	1	2	3	4	5	6	7	8	9	10
Dodd et al., 2014 [39]	+	+	+	+	+	+	+	+	+	+
Dodd et al., 2016 [41]	+	+	+	+	+	+	+	+	+	+
Manthorpe et al., 2003 [12]	+	+	+	+	+	?	?	+	+	+

+ Yes, - No, ? Unsure

Criteria

1. Was there a clear statement of aims?

2. Is a qualitative methodology appropriate?

3. Was the research design appropriate to address the aims of the research?

4. Was the recruitment strategy appropriate to the aims of the research?

5. Was the data collected in a way that addressed the research issue?

6. Has the relationship between the researcher and participants been adequately considered?

7. Have ethical considerations been taken into consideration?

8. Was the data analysis sufficiently rigorous?

9. Is there a clear statement of findings?

10. Does the research make a contribution to existing knowledge or understanding?

**Table 3:** Risk of bias summary. Mixed Methods studies. Mixed Methods Appraisal Tool (MMAT) Version 2018 [46]

Criteria					
Studies	1	2	3	4	5
Iliffe et al., 2014 [35]	+	+	+	+	+
Millard et al., 2011 [3]	+	+	+	NA	_
Ollerenshaw et al., 2017 [32]	_	_	+	?	_
Perry et al., 2010 [34]	+	+	+	+	+

+ Yes, - No, ? Can’t tell

Criteria questions

1. Is there an adequate rationale for using a mixed methods design to address the research question?

2. Are the different components of the study effectively integrated to answer the research question?

3. Are the outputs of the integration of qualitative and quantitative components adequately interpreted?

4. Are the divergences and inconsistencies between qualitative and quantitative results adequately addressed?

5. Do the different components of the study adhere to the quality criteria of each tradition of the methods involved?

**Table 4:** Risk of bias summary. Survey studies. Critical Appraisal of a Survey checklist [45]

Criteria												
Study	1	2	3	4	5	6	7	8	9	10	11	12
Gilbert et al., 2017 [29]	+	+	+	+	+	_	_	+	+	+	+	_
Manthorpe et al., 2003 [12]	+	+	+	+	_	_	+	+	+	+	?	_
Trickey et al., 2000 [11]	+	+	+	_	+	_	+	?	_	_	+	_

+ Yes, - No, ? Can’t tell

1. Did the research study address a clearly focused research question?

2. Is the study design appropriate for answering the research question?

3. Is the methods of selection of the subjects clearly described?

4. Could the way the sample was obtained introduce selection bias?

5. Was the sample representative of the population to which the findings will be referred?

6. Was the sample size based on pre-study considerations of statistical power?

7. Was a satisfactory response rate achieved?

8. Are the measurements (questionnaires) likely to be valid and reliable?

9. Was the statistical significance assessed?

10. Are confidence intervals given for the main results?

11. Could there be confounding factors that haven’t been accounted for?

12. Can the results be applied to other settings?

### Synthesis of results

Synthesis of data from studies so diverse in research questions, methodologies, nurse scope of practice and health systems is inherently problematic and it was not possible to sensibly categorise findings into themes.

**Fig. 2:**
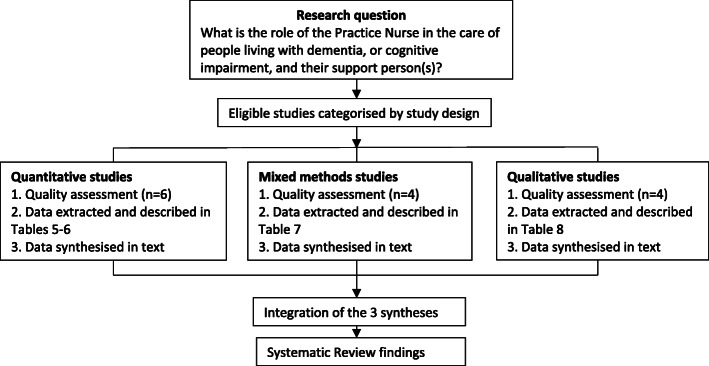
Stages of the review

In this systematic review a rigorous and transparent method was utilised to organise, describe, explore and interpret the findings and generate new insights [48, 49]. Eligible studies were selected using the defined inclusion criteria and then categorised into groups according to study design. Quality and risk of bias assessment was carried out according to their specific study types. Following quality assessment, data were extracted from the studies and tabulated under the headings: research aim; study design; instruments; sample characteristics; intervention type; analysis and outcomes. (Tables 5, 6, 7 and 8). The data were synthesised according to the three study types; quantitative, qualitative and mixed methods. The three syntheses were then integrated into one synthesis which informed the findings of this systematic review. (Refer Fig. 2).

**Table 5:** Quantitative studies. Characteristics of the Randomised Controlled Trials reviewed

Author, year, country	Aim	Research design	Instruments	Sample and size (+characteristics)	Type and description of intervention	Analysis method	Outcomes
Van den Dungen et al., 2016 [15] The Netherlands, United Kingdom Supplementary papers: Perry et al., 2008 [16] Van den Dungen et al., 2012 [17]	To assess the effect of a two-component intervention of case finding and subsequent care on diagnostic yield of case finding and its impact on the mental health of patients and relatives.	Cluster randomised controlled trial with process evaluation	Cambridge Cognition Examination (CAMCOG) Quality of Life-Alzheimer’s Disease (QoL-AD) Mental Health part of SF-36 (MH5) Short form health survey (SF36) MDS Euro-Qol (EQ5D) 12-item General Health Questionnaire (GHQ12) Short sense of Competence Questionnaire (SSCQ) Neuropsychiatric Inventory (NPI) 12-item Social Support List (SSL12) 15-item Katz questionnaire (Katz15) Mini Mental State Examination (MMSE)	162 participants ≥ 65 years in 15 primary care practices in whom Family Practitioners (n=29) suspect cognitive impairment, but without a dementia diagnosis 2 PNs over 7 intervention practices	Intervention 1: Family practitioners (FP) attended 5 hours of dementia education. Education content based on the EASYcare dementia training program described in Perry et al., [18] Intervention 2. In addition to above, case finding of MCI and dementia and collaborative care by a dementia trained PN (a Registered Nurse) and the FP. Nurse assessment and care planning was based on the outcomes of the Residential Assessment Instrument (RAI).	Generalised Estimating Equations (GEE) analysis Odds Ratio, 95% confidence interval	Training FPs resulted in a non-significant increase in the number of new MCI diagnosis. There were no differences in mental health (QOL measure) between the group receiving collaborative care and the control group. FPs and PNs found care management to be a positive experience, although the nurses it to be time consuming. Further study of collaboration between FP and PNs is recommended.
Thyrian, J.R.et al., 2017 [19] Germany. Supplementary papers: Dreier et al., 2016 [20] Thyrian et al., 2013 [21].	To test the effectiveness and safety of Dementia Care management (DCM) in the treatment and care of people with dementia living at home and caregiver burden (when available)	Cluster-randomised intervention trial	Quality of Life (QoL-AD score) Neuropsychiatric symptoms (NPI score) Caregiver burden (BIZA-D score) Anti-dementia drug treatment Potentially inappropriate medication prescription	634 people diagnosed as having dementia 407 received the intervention	DCM Is a model of collaborative care aimed at providing optimal care for patients with dementia and support care-givers. DCM uses a computer-assisted assessment determining a personalised array of intervention modules and monitoring. DCM was provided by a dementia trained RN for 6 months in the home according to a systematic, detailed protocol. The nurses completed an intensive training program described in Drier et al., [21].	Descriptive statistics - Means, SD - Proportions Primary analysis - Generalised regression models Secondary analysis - Stratification of the models by patient’s living situation	A significant decrease in patient’s behavioural and psychological symptoms of dementia and caregiver burden was reported. There was a significant increase in quality of life for patients not living alone, but no improvement in quality of life overall. No significant effect on patient’s cognitive status, daily living activities, or institutionalisation was found.
Callahan et al., 2006 [18] United States Supporting papers Austrom et al., 2005 [22] Austrom et al., 2006 [23] Austrom et al., 2004 [24] Boustani et al., 2005 [25]	To test the effectiveness of a collaborative care model to improve the quality of care for patients with Alzheimer’s disease.	Randomised Controlled Trial	Total patient Neuropsychiatric Inventory (NPI) Total caregiver Neuropsychiatric Inventory (NPI) Cornell Scale for Depression in Dementia (CSDD) Telephone Interview for Cognitive Status Patient Health Questionnaire-Alzheimer Disease Cooperative Study Group ADLS Caregiver Patient Health Questionnaire-9	153 predominantly ethnic older adults with Alzheimer Disease and their care-givers 84 people received the intervention of collaborative care management The settings were large primary care practices, community-based health centres and a Veteran Affairs Medical Centre.	12 months of care management using current Alzheimer’s Disease treatment guidelines Delivered by a FP and an advanced PN (geriatric nurse practitioner) acting as the care manager Care-givers and patients were seen by the nurse fortnightly and then monthly for period of 1 year There were 4 intervention components 1. A behavioural intervention protocol (described in Austrom et al., [24], Boustani et al.,[25] 2. Weekly Care manager support meetings 3. A web-based longitudinal tracking system 4. voluntary group care-giver sessions for care-givers with a group exercise group for patients	2-tailed α level of 0.05 2-sample t tests Χ^2^ tests Mixed-effects regression Kaplan-Meier estimation Wilcoxon test	Patients experienced significant improvements in total NPI scores which continued beyond the 12 month intervention The intervention had no significant impact on patient depression scores, cognition or function. There were significant improvements in caregiver stress at 12 months but not at 18 months. There was no difference in cumulative hospitalisation rates , mean hospital days or rates of nursing home placement

**Table 6:** Quantitative studies. Characteristics of the Survey/ Questionnaire studies reviewed

Author, year, country	Aim	Research design	Instruments	Sample and size (characteristics)	Type and description of intervention	Analysis method	Outcomes
Trickey et al., 2000 [11] United Kingdom	To examine the knowledge and attitudes of primary care nurses who undertake the Over-75 Check, towards assessing and managing patients with symptoms of dementia, and to assess their level of support for a clinical practice guideline.	Descriptive	Postal questionnaire survey of primary care nurses responsible for the Over-75 Check Questionnaire included -a case vignette for eliciting information about knowledge, attitudes and opinions -demographic information	127 (65% response rate) respondents -71% practice based nurses -11% health visitors -6% district nurses -12% other All respondents were female 75% over 40 years 32% over 50 years 18% had completed a post-registration course in nursing older people		Analyses using SPSS 6.0 and the commands ‘descriptives’ and ‘crosstabs”	In response to the vignette PN were less likely to take any action themselves than were other professionals (61 vs 78%) (just failed to reach statistical significance (chi-squared test P=0.07) Thematic analysis of the vignettes revealed that the nature of the referral to the GP was influenced by the respondent’s knowledge of dementia, understanding own professional role and by structural constraints such as the need to use the GP to access other services. It was reported that many professionals undertaking the Over-75 Check are not sufficiently well trained to assess patients who may be displaying signs of cognitive impairment 68% of respondents indicated that new guidelines would ‘definitely help’ them in their practice.
Manthorpe et al., 2003 [12] United Kingdom Supplementary papers Downs & Rae, 1996 [26] Iliffe et al., 1999 [27] Illife et al., 2003 [28]	To explore whether Community Mental Health Nurses (CMHNs ), Community Nurses (CNs), and PNs have different perspectives on early diagnosis of dementia	Intervention Comparative	Questionnaire derived from the Stirling Service Development Centre asked the nurses about clinical role, experience, case load, epidemiological and clinical knowledge, confidence in recognising the dementias and perceived difficulties in providing care for people with dementia	268 nurses (79 CMHNs, 153 CNs, 36 PNs)	1 day educational workshop on recognition of and response to dementia	Data was aggregated by professional discipline 95% confidence intervals 1.96 x √*(p x q/n)* *P* proportion showing the characteristic *Q* proportion not showing the characteristic *n* is the sample size	All three nurse disciplines reported experience of working with people with dementia and had similar knowledge related the early signs and symptoms of dementia. However CMHNs were more confident in their diagnostic skills, ability to communicate a diagnosis, provide advice and obtain support services. CMHNs considered they were best placed to coordinate services for people with newly diagnosed dementia and found providing support less difficult than CNs and PNS. Dementia support in the community was seen as a specialist function, with the key worker role best fulfilled by CMHNs.
Gilbert et al., 2017 [29] United Kingdom Supplementary papers Downs et al., 2006 [30] Illife et al., 2010 [31]	To explore the service use and reported unmet needs of people with dementia recruited a decade apart	Questionnaire Comparative	The questionnaire covered demographics and capabilities of the person with dementia as perceived by the support person(s) assessed across nine different activities of daily living	The 2 samples were recruited as part of 2 previous RCT studies [30, 31] Both samples have similar demographics and the people with dementia had similar degrees of disability and engagement with community services. Sample 1 Central Scotland and London 2000-2001 Support person(s) = 122 Sample 2 South-East England 2010-2011 Support person(s) = 84		Summary descriptive statistics One-tail chi-square Fisher-exact analysis Logistic regression Binary linear regression analysis	There were significantly more support person(s) who contacted PNs about the person with dementia in the later sample than the earlier (53.6% compared with 36.9**%,** p=0.01) and less evident use of CNs, psychiatric nurses and health visitors. PN use was associated with having a non-spouse support person(s) (p=0.022) but no other factors were significant. Those who received PN help generally found it useful. Support person(s)s in the second sample reported that approximate one third of people with dementia were still not getting the support they needed and the majority of support person(s)s reporting behavioural and psychological problems had had no advice on how to manage this

**Table 7:** Mixed Method studies. Characteristics of Mixed Methods studies reviewed

Author, year, country	Aim	Research design	Instruments	Sample and size (+characteristics)	Type and description of intervention	Analysis method	Outcomes
Ollerernshaw et al, 2017 [32] Australia	To explore the awareness and usage of an online Dementia Pathways Tool (DPT) for primary health care practitioners in regional Victoria	Descriptive	On-line questionnaire Google Analytics provides information on the usage of on-line tools.	A total of 21 GPs and 21 PNs participated in the study. All the GPs and PNs worked in practices located in regional western Victoria.		Descriptive analysis	Of the respondents two thirds were aware of the DPT, with one third having used it. Of those who had used it the majority (92.9%) found it useful in assisting them in accessing diagnosis, management and referral information. Over an 18 month time period from the launch of the DPT, Google Analytics showed an average of 509 views of the DPT webpage a month with an average of 2.03 minutes spent on the site.
Millard et al., 2011 [3] Australia Supplementary paper Millard & Baune, 2009 [33]	To explore dementia literacy in a general practice setting and to test whether a waiting room pamphlet would improve patient awareness of dementia risk reduction	Mixed method study: questionnaire, RCT and data from computerised medical records	Questionnaire for GP/ PN Questionnaire for waiting room patients Computerised medical records.	558 patients(36% male 64% female; median age 50-59 years) were sampled from 22 practices in 14 locations in Australia 63 patients (43% male; 57% female; median age of 70-79 years) were sampled from 3 practices in England. 106 GPs (57 males; 49 female) were recruited from 50 different sites in Australia and 21 GPs (9 male; 12 female) were in England. 26 PNs were recruited to the study in total. All were female and 25 were located in practices in Australia and 1 in England.	Alzheimer’s Australia ‘Mind your Mind” dementia risk reduction pamphlet on patient knowledge of dementia risk strategies Patients, aged over 30 years, in the waiting room were administered a questionnaire exploring dementia and risk reduction knowledge. The intervention, a risk reduction education pamphlet, were administered to the patients using a simple randomisation with 50% of the participants receiving the pamphlet with questionnaire and 50% receiving the questionnaire only.	SPSS Bivariate analysis Pearson’s Chi-squared test Odds ratio	There were no significant differences in dementia literacy between age groups, country or gender. Sources of dementia knowledge included ‘media’ (32.5%), ‘acquaintances’ (30.6%), and ‘workplace’ (15.9%) with a minority answering ‘doctor’ (1.3%). Despite the majority of respondents having not heard about dementia from their doctor, 81% would seek help from a doctor if thought they had dementia. One third of GPs and two thirds of PNs reported lacking dementia training. Just under one quarter of GPs and under one fifth of PNs considered their dementia knowledge adequate. There was no significant relationship between training and adequacy of dementia knowledge. Despite this lack of self-reported lack of dementia knowledge, two thirds of doctors and three quarters of PNs responded that a doctor or nurse was the appropriate person to discuss dementia with patients.
Perry et al., 2010 [34] Netherlands	To construct a set of quality indicators (QIs) for dementia diagnosis and management in a primary care setting.	Mixed methods RAND modified Delphi including a postal survey, stakeholders consensus meeting, a scientific expert consensus meeting and demonstration project	Postal survey GPs and PCNs assessed relevance, feasibility of QIs Informal support person(s) assessed relevance of QI only Inclusion, exclusion, revision of QIs by consensus at stakeholder and scientific expert meetings Demonstration project tested adherence rates and discriminative validity.	GPs (67), PCNs (21) and informal care-givers (34). Eight national dementia experts (1 geriatrician, 2 GPs, 2 nursing scientists, 1 medical sociologist, 1 psychologist, 2 geriatric nurses) in expert panel and scientific consensus meeting. 1 GP, 2 PCNs and 4 informal support person(s) in stakeholders consensus meeting Thirteen GPs in the demonstration project. GPs and PCNs were recruited at continuing medical education meetings. Informal support person(s) were recruited at the memory clinic of the Radboud University Nijmegen Medical Centre.		Cronbach alpha Intraclass correlation Mean scores and standard deviations ANOVA	The final set of 23 QIs included 15 QIs containing innovative quality criteria on collaboration between GPs and PCNs, referral criteria and assessment of support person(s) needs. Several indicators explicitly describe collaboration between GPs and PCNs, an area in which improvement is highly recommended. The QIs are reported as feasible, reliable and valid able to be used to improve primary dementia care.
Iliffe et al., 2014 [35] United Kingdom Supplementary papers Bamford et al., 2014 [36] Iliffe et al., 2014 [37] Waugh et al., 2013 [38]	To adapt a US model of primary care-based case management (CM) (PREVENT) for people with dementia and test it in General Practice	Mixed methodology comprising case studies of CM implementation in four General Practices and Interview with patients, support person(s), local NHS and other stakeholders, and case managers	CAREDEM Case management	Participants were community dwelling patients with dementia who were living at home with a family support person(s) and who were not receiving specialist care coordination. A total of 28 dyads and 1 support person(s) were recruited across four practices; one rural, one inner-city and two urban.	The CAREDEM intervention consisted of CM face to face training and mentoring based on an educational needs assessment, development of a learning manual that could be shared between CMs and patient-support person(s) dyads and identification of skills needed by dementia CMs working in primary care.	Thematic analysis of data was synthesised in an iterative process until consensus was reached	This study suggested that CM does not fit easily into practice routines and that it was not substantially beneficial for patients and support person(s) despite CMs, patients and support person(s) reporting a positive experience of CM. Perceived benefits of CM by the dyad included having a first point-of-contact, a ‘safety net’ and the creation of a one-to-one therapeutic relationship. CM perceived advantages was provision of continuity of care and flexibility of responsiveness to needs. The NHS and social care professionals perceived advantages of CM as continuity of care, earlier intervention and that it was complementary to existing secondary care and social services. All case managers cited time constraints as an obstacle to working with their target group and identified relatively few concrete benefits to participating patients and support person(s).

**Table 8:** Qualitative studies. Characteristics of the qualitative studies reviewed

Author, year, country	Aim	Research design	Instruments	Sample and size (+characteristics)	Type and description of intervention	Analysis method	Outcome
Dodd et al., 2014 [39] United Kingdom	To contrast participant’s experiences of primary care led dementia services in Bristol with existing secondary care based memory services	Qualitative participatory study	Semi-structured interview. Questions were organised under four main themes (1) GPs making an independent dementia diagnosis (2) GPs working with memory nurses (3) patients and support person(s) experience, and (4) post-diagnostic support. The interviews lasted, on average, 20 minutes.	Purposive. A total of 23 participants (10 health care professionals, 6 patients and 7 support person(s)). Eligible patients were people of any age who had been diagnosed within the previous six months with any form of dementia (MCI excluded) Participants were recruited from GP practices in Bristol offering primary care led dementia services and secondary care health services GPs were invited by email to participate in the study. Health care professionals in secondary care were recruited through team meetings.	A pilot model of primary care led dementia care with three memory nurses seconded from secondary care to work within primary care with each memory nurse providing an advisory service to a group of practices. Participating GPs attended a three-hour training session on identifying, assessing and diagnosing dementia.	Data coding and identifying evidence that related to each of the four themes from the panel meetings using a prescribed process, described by Clarke [40].	Patients and support person(s) were uniform in the praise of the work by memory nurses in both primary and secondary care settings. Many GPs did not feel confident to conduct a dementia assessment without involving a memory nurse and reported that they valued the working relationship with the memory nurse. Memory nurses found liaising with GPs to be cumbersome and time consuming. Patients and support person(s) perceived there was an absence of supporting patients through the assessment and post diagnostic support from GPs. Information came from memory nurses and other memory service staff or through the media.
Dodd et al., 2016 [41] United Kingdom	To provide a qualitative analysis of the experiences of health care professionals, patients and their families, of the new process of assessment, diagnosis and treatment of dementia within a primary care service.	Interview Descriptive	Semi-structured interview	23 surgeries in the South Gloustershire Four patients, three care-givers and eight health care professionals were interviewed by peer researchers (care-givers).	Primary care led dementia service in which GPs led the process of assessment and establishing a diagnosis. A memory nurse (from secondary care) was available to support GPs and patients and support person(s) with diagnostic process.	Thematic analysis using Braun and Clarke [42] six-phase model	There were two consistently expressed concerns by all groups of participants (1) lack of post-diagnostic co-ordination (2) GP-led or multi—disciplinary assessment without secondary care support. The advisory role of the memory nurse in the primary care service was valued by GPs. Referral to secondary care memory nurses who provided specialised assessment and post-diagnostic support, including home visits, was valued by patients and support person(s).
Manthorpe et al., 2003 [43] United Kingdom Supplementary papers Iliffe et al., 1999 [27] Iliffe et al., 2003 [28]	To explore the implications of the early recognition of dementia for inter-professional working.	Focus group interviews	Nominal group technique	Convenience sample. Nearly 1000 GPs (247), CMHNs (79), practice (36) and CNs (146), social workers and nursing home staff took part in four focus groups in each of 24 multi-disciplinary workshops in the UK .		Thematic and interpretative analysis of data	Four key bipolar categories were derived from the workshops. (1) opportunistic versus population screening (2) referral and responsibility (3) key working and team working (4) generalist versus specialist roles. Nurses overall were the professional group considered to have the skills and capacity in dementia care with CMHNs as specialists who may take on long-term and complex cases. The PN was identified as the practitioner most appropriate to take on screening for dementia and monitoring. It was reported that screening by the PN become a core skill and routine in primary care.

This approach provided an analysis of the published academic literature and enabled the exploration of relationships within and between studies and a description of themes across the included studies.

## Results

The search strategy identified 1870 references (Fig. 1). After removal of duplicates 1802 abstracts were examined for relevance and 68 full text references were obtained for full text screening. Hand-searching of references lists of included articles yielded an additional three articles. In total 71 articles were assessed for eligibility, of which 13 articles were selected for data extraction and analysis.

Fifty-eight studies assessed for eligibility were excluded. Eighteen were grey literature, 17 did not include the primary health care nurse, six were poster abstracts and the studies not published, and 17 papers were removed as they were multiple publications reporting on the same intervention and were included as supplementary papers. Three were duplicate studies [18, 22, 23] and two studies [16, 50] were excluded as the outcomes had not been published. The authors of these studies were contacted. Bryans et al., [50] did not publish the outcomes of a survey study on primary health care nurses and dementia care due to significant loss to follow-up. For similar reasons, Perry et al., [16] did not publish the outcomes of the dementia training programme on diagnostic assessment and management of dementia by primary care nurses.

### Study characteristics

Of the 13 included studies, six were quantitative studies: three RCTs and three survey questionnaires, four were mixed-method studies and three were qualitative studies using interviews.

The studies were conducted in the Netherlands (n=1), Germany (n=1), United States of America (n=1), The United Kingdom (n=5), Australia (n=4) and one was conducted across the Netherlands and the United Kingdom (n=1).

Four studies [15, 18, 19, 35] evaluated dementia care management in primary health care. Exploring dementia care knowledge and attitudes of primary health care practitioners was the focus of three studies [3, 11, 12]. Two studies [39, 41] explored participant experiences of dementia care delivery in primary health care and one study [29] explored service use and reported unmet needs of people with dementia and support person(s). Investigating the implications of early recognition of dementia for the roles of the primary health care team was the focus of one study [43] The authors of one study [34] developed quality indicators for dementia care in primary health care settings and one study investigated the value and useability of an online dementia management tool for health professionals [32]. The study interventions and outcomes are described in Tables 5, 6, 7 and 8.

#### Quantitative Studies

##### Randomised Controlled Trials

Three studies utilised an RCT [15, 18, 19] to investigate the impact of collaborative care on quality of life for people with dementia and their caregivers. The study by Van den Dungen et al., [15] also included an evaluation of family practitioner training on diagnosis of mild cognitive impairment.

In all three models of care the nurse was the care manager who worked in collaboration with the primary care doctor. All care management models followed a structured assessment and care planning protocol. Care management ranged in duration from six [19] to twelve months [15, 18]. In two studies [15, 19] the care managers were registered nurses, with Van den Dungen et al., [15] specifying the nurse as a primary care nurse who acted as the study nurse. In the third study [18] the care manager was a geriatric nurse practitioner. All the nurses received dementia specific training and were integrated into the primary care team with only one care manager providing the dementia care management within the patients’ home [19]. In addition to training, in the model of care described in Callahan et al., [18] the nurse received weekly support from a geriatrician, geriatric psychiatrist and a psychologist.

Callahan et al., [18] and Thyrian et al., [19] reported a significant decrease in behavioural and psychological symptoms of dementia and caregiver stress with dementia care management, however, Thyrian et al., [19] reported there was no significant improvement in quality of life overall. Despite reporting that dementia care management had no impact on quality of life measures for patients or their care-givers, Van den Dungen et al.,[15] recommend that collaborative care with nurses in primary care deserves further exploration.

##### Survey Questionnaire studies

Three studies reported survey results [11, 12, 29]. Manthorpe et al., [12] and Trickey et al., [11] investigated dementia knowledge and attitudes of community nurses (CN), health visitors, community mental health care nurses (CMHN) and PNs in the provision of care for people living with dementia. The third study [29] explored service use and unmet needs of people with dementia recruited a decade apart.

Manthorpe et al., [12] reported all groups of primary health care nurses had similar knowledge related to the early signs and symptoms of dementia. However, PNs were less confident in providing advice and support than CMHNs. In the study undertaken by Trickey et al., [11], PNs completing the Over-75 year health check were less likely than other nurse groups to take any action, other than to refer to the GP, when presented with a person living with dementia and their support person. The Over-75 year health check is an annual health check including a mental assessment for people aged over 75 years [11].

Gilbert et al., [29] reported that support person(s) were increasingly contacting a PN for support with less evident use of CNs, health visitors and CMHNs. This may in part be attributed to greater access to a PN and the changing nature of the PN role with an increased focus on chronic disease management. Support person(s) reported that they were still not getting the advice and support they needed.

Authors of all three studies identified a need to improve PN knowledge of dementia and its management. In the study by Trickey et al., [11] participants reported guidelines would be helpful to address gaps in knowledge and to standardise practice.

#### Mixed method studies

Four studies reported mixed-methods research results [3, 32, 34, 35].

Perry et al., [34] used a RAND modified Delphi method to construct a set of quality indicators for dementia diagnosis and management in primary care in the Netherlands. PNs were involved in the selection and validation process of the quality indicators. Of the final 23 quality indicators, two explicitly describe collaboration between the GP and the PN, an area in which the authors suggest improvement is highly recommended. A further three quality indicators emphasise the importance of developing and reviewing individualised care plans. This is commonly a PN role that is established and accepted in primary care settings [34]. Millard et al., [3] explored dementia literacy in a general practice setting. In this study two-thirds of the PNs reported a lack of dementia training. Despite this self-perceived lack of training, three-quarters of the PNs reported that the primary care doctor or nurse was the appropriate person to discuss dementia with patients. Ollerenshaw et al., [32] suggest that PNs may find an on-line dementia management support tool useful. Iliffe et al., [35] adapted a US model of primary care based care management (PREVENT) for people with dementia and tested its implementation in UK general practice. Despite case managers, patients and support person(s) reporting a positive experience and perceiving benefits of case management, Iliffe et al., [35] suggest that case management does not fit easily into practice routines and that it was not substantially beneficial for patients and support person(s).

#### Qualitative studies

All three qualitative studies [39, 41, 43] used interviews to explore experiences of primary health care practitioners, patients and support person(s), of dementia care. Dodd et al., [39] used semi-structured face-to-face interviews to contrast study participants’ experiences of a new primary care led dementia service with existing secondary care based memory services in Bristol, UK. Dodd et al., [41] used a semi-structured face-to-face interview to investigate participant’s experiences of a new primary care led dementia service in South Gloustershire, UK. In both these studies [39, 41] the nurses were seconded from secondary care dementia services, with each nurse working with a group of primary health care clinics. Patients and support person(s) reported primary care led services to be positive and there was uniform praise for the work by the memory nurse. GPs reported they valued the advisory role provided by the memory nurse. Manthorpe et al., [43] explored implications of the early recognition of dementia for inter-professional working using focus group interviews. In this study the PN was identified as the practitioner most appropriate to take on screening for dementia and monitoring, however community mental health care nurses were considered to have the skills and capacity to take on long-term and complex cases.

##### Risk of bias

The methodological quality varied across the studies (Tables 1, 2, 3 and 4). The qualitative studies and all but one of the mixed methods studies rated high according to the quality appraisal criteria. Of the quantitative studies two of the three RCT studies lacked allocation concealment, blinding and presented incomplete outcome data which compromised their quality. The survey studies were of mixed quality with two of the three studies introducing selection bias and no sample size was based on consideration of statistical power.

In addition to these limitations, Callahan et al., [18] describe their study as unable to identify which of the subcomponents of the intervention were most effective in achieving the outcomes. Van den Dungen et al., [15] reported the rates of MCI or dementia identified were lower than expected. The authors state the reasons for this may have included a type 2 error with a low sensitivity of the cognitive tests performed by PN. In addition, there was sub-optimal implementation of the intervention with the family practitioner not always performing further diagnostic assessments on all persons referred by the PN [15]. Thyrian et al., [19] describe limitations of the study including potential selection bias as screening and recruitment were part of routine care. The intervention and control groups had an uneven number of participants; the GPs in the control group had fewer patients. In addition, the GPs may have become aware of their assignment to the control or intervention group [19].

Trickey et al., [11] describe a methodological limitation of using a vignette that may more correctly explore current practice rather than knowledge and attitudes [11]. Iliffe et al., [35] report time constraints for the case management role of the PNs may have meant there was insufficient time to show the potential of case management.

## Discussion

This systematic review of the published literature, available in English, on the current and potential role of the PN in the delivery of care to people living with dementia or cognitive impairment and their support person(s) evaluated thirteen studies.

There has been no previous systematic reviews of the role or potential role for the PN in the delivery of care to people living with dementia or cognitive impairment and their support person(s). The results from this review are therefore novel and should be used to inform the role of the PN in the provision of dementia care and also future research on this topic.

The heterogeneity of studies’ purpose, design, and outcomes measures make it difficult to synthesise the findings and draw conclusions. However, the heterogeneity did provide important insights into the different roles of nurses and advances understanding about the intervention itself rather than just its effectiveness. The only clearly defined role that was examined was that of the primary care based nurse as a care manager [15, 18, 19, 35]. There were mixed findings regarding the effectiveness of the nurse-led care management model of care in improving quality of life measures for people living with dementia and their support person(s). However, no studies dismissed the potential of this model, with further research recommended. Callahan et al., [18] was assessed as the highest quality RCT study. The authors reported that a care management model of care can be implemented in primary care and that the effectiveness of the intervention depended on the key role of the nurse. All the nurses in these care management studies were registered nurses with dementia specific training, however in the Callahan et al., [18] study the care manager was a geriatric nurse practitioner. All health practitioners in the care manager studies described the experience as positive and perceived there to be benefits to the patient. Nurses did describe the role as time consuming and liaising with the primary care medical practitioner as cumbersome [15, 39]. However, the care manager role was considered resource intensive, which could prove a challenge in its integration with practice routines that often operate, with limited time for consultations and budgetary constraints. The care management model described in Callahan et al., [18] was particularly resource intensive with one year of care management, weekly mentoring for the care manager, weekly then monthly patient contacts, and monthly care-giver support groups with concurrent exercise groups for the person living with dementia.

The other studies [3, 11, 12, 29, 32, 34, 39, 41, 43] explored characteristics of the role of the primary care based nurse in the care of people living with dementia and the support person. These studies were of variable quality but consistent in their outcomes. The PN was described as having an increasing profile in primary health care and being more accessible to patients, partly as a result of their changing role to include chronic disease management. There was recognition of the PN as the appropriate professional to take on the role of screening for cognitive impairment and monitoring, with the medical practitioner being responsible for diagnosis. The PN is usually responsible for the Over 75 health check which is currently underutilised [11] and provides an opportunity to identify people with cognitive impairment. A common issue in the studies was the poor recording of diagnosis or outcome of cognitive testing in electronic medical records. Several studies identified that post-diagnostic support and carer support were lacking in current dementia care provision in primary health care [29, 35, 39]. Patients with memory concerns reported that they would welcome the opportunity to discuss dementia risk reduction with the GP however the GP was not meeting this need [3]. This responsibility was reported as potentially within the scope of the primary care nurse role [3].

Developing good working relationships with the medical practitioner, familiarity with the primary care setting, perception of autonomy, dementia specific education and the embedding dementia care provision in primary health care were seen as essential to the success of the primary care nurse in dementia care provision. A consistent finding across the studies was that primary care nurses reported a lack of confidence in dementia care provision and the rating of their knowledge and skills as inadequate. This is despite the perception that nurses include themselves as an appropriate professional to discuss dementia with a patient. The need for education and training was stressed in all studies as necessary for successful dementia care provision. The use of guidelines was perceived as valuable by nurses to improve knowledge and standardise practice. Nurses in the care management models used detailed standardised protocols for dementia care provision.

### Implications for practice and research

There is justification for the involvement of the PN in the recognition and care of people living with dementia and their support person(s). However, there is little evidence on the scope of practice and framework of primary care nurse models of dementia care provision. The different studies examined different aspects of the PNs role in relation to dementia. Differences in scopes of nurse practice and health systems mean one model of care may not be appropriate. However this systematic review provides insights into what components of a model of care may be effective. These roles included care management, identification and/ or management of behavioural and psychological symptoms of dementia. Some nurses were seconded from secondary care memory clinics, some were registered nurses working in general practice and one was a geriatric nurse practitioner. Dementia training for the nurses also greatly varied across studies from several hours to months and the types of training differed in breadth and intensity.

More high quality studies are required to establish the scope of practice, effectiveness, cost implications and the applicability of the PN role in the care of people living with dementia, or cognitive impairment, and their support person(s) in general practice.

### Strengths and limitations

This is the first systematic review to investigate the role of the PN in the care of people living with dementia, or cognitive impairment, and their support person(s) in general practice. An explicit, systematic methodology was followed to review the published peer-reviewed literature relevant to the topic. National and international literature was reviewed and the studies utilised a variety of methodologies including qualitative, quantitative and mixed methods. It was not possible to conduct a meta-analysis due to the heterogeneous nature of the interventions. The studies included in this review were published in English only and grey or white literature was not included. Some studies may not have been identified by the search terms used in each database.

## Conclusions

The aim of this systematic review was to investigate the role of the PN in the care of people living with dementia, or cognitive impairment, and their support person(s) in general practice. The potential value of the PN in the recognition and management of dementia has been acknowledged. However, the findings of this review revealed that there is limited evidence on the role of the PN in dementia care provision. The strength of this review is the identification of benefits of roles fulfilled by nurses in the general practice setting for people living with dementia and their support person(s). These included increased patient accessibility to the PN, early recognition and management of cognitive changes, care management and collaboration with the GP. Limitations of the provision of dementia care by the PN included a lack of definition of the role, inadequate dementia specific training, time constraints and poor communication with GPs.

Models of dementia care provision with mechanisms to support the practice nurse role and the embedding of it into usual general practice care have the potential to increase early recognition of cognitive impairment and more appropriate primary care management of dementia.

## Data Availability

Not applicable.

## Abbreviations

APNA: Australian primary health nurse association
CMHN: Community mental health nurse
CN: Community nurse
DCM: Dementia care management
FP: Family practitioner
GP: General practitioner
MMSE: Mini mental state examination
NHS: National health service
QOL: Quality of life
RCT: Randomised controlled trial
